# Upregulation of D site of albumin promoter binding protein in the brain of patients with intractable epilepsy

**DOI:** 10.3892/mmr.2014.3065

**Published:** 2014-12-09

**Authors:** JINXIAN YUAN, JING GUO, MELIN ZHANG, QIAN WANG, HAO HUANG, YANGMEI CHEN

**Affiliations:** Department of Neurology, The Second Affiliated Hospital of Chongqing Medical University, Chongqing 400010, P.R. China

**Keywords:** intractable epilepsy, temporal lobe, D site of albumin promoter binding protein, mitogen-activated protein kinases

## Abstract

The mechanisms that underlie the pathogenesis of intractable epilepsy (IE) remain to be elucidated. The present study aimed to investigate the expression of D site of albumin promoter binding protein (DBP) and mitogen-activated protein kinases (MAPKs) in the temporal lobes of patients with IE, in order to examine the possible roles of DBP in the pathogenesis of IE. The expression of DBP and MAPK was detected by immunohistochemistry and double-label immunofluorescence staining against DBP/MAPK in 35 patients with IE, and the data were compared with those of the 15 controls. The results demonstrated that DBP expression in IE group (0.31±0.03) was significantly higher compared with that in the controls (0.18±0.02; P<0.05) and MAPK expression in the IE group (0.19±0.03) was also higher compared with that in the controls (0.12±0.02; P<0.05). DBP and MAPK were mainly expressed in the cytoplasm of neurons and the double-label immunofluorescence staining demonstrated that DBP and MAPK expression occurred in the same neurons. Therefore, the expression of DBP and MAPK in epilepsy patients was upregulated, suggesting a possible pathogenetic role in IE.

## Introduction

Epilepsy is a chronic disease characterized by recurrent episodes of abnormal high-frequency neuronal discharge resulting in the transient disturbance of cerebral function. ~80% of all epilepsies are well-managed with drugs and numerous patients have prolonged remissions. The remaining 20% are medically intractable, often resistant to treatment with various antiepileptic drugs (AEDs) at maximal dosages as monotherapies or in combination, and are thus referred to as intractable epilepsy (IE) cases (1). The underlying mechanisms of IE remain elusive and represent a highly challenging topic of epilepsy research that has attracted notable attention in recent years.

D site of albumin promoter binding protein (DBP), hepatic leukemia factor (HLF) and thyrotroph embryonic factor (TEF) are the three members of the proline and acidic amino acid-rich basic leucine zipper (PAR bZip) transcription factor family. All three of these transcriptional regulatory proteins accumulate with robust circadian rhythms in tissues with high amplitudes of clock gene expression, including the suprachiasmatic nucleus (SCN) and the liver. In mammals, the circadian timing system controls numerous aspects of behavior and physiology, including rest-activity cycles, heartbeat frequency, body temperature, arterial blood pressure, endocrine functions, renal plasma flow, intestinal peristalsis and metabolism (2). PAR bZip proteins control the expression of numerous enzymes and regulators involved in detoxification and drug metabolism, including cytochrome P450 enzymes, carboxylesterases and constitutive androstane receptor (CAR) (3). Only DBP is capable of efficiently activating transcription from the cholesterol 7α-hydroxylase (C7αH) promoter (4). It has been suggested that DBP may have an important role in drug resistance.

However, DBP expresses at a nearly invariable level in the majority of brain regions, in which clock gene expression only cycles with low amplitude (4,5). Klugmann *et al* (6) reported that DBP expression gradually increased between postnatal day 1 and day 60. Several studies have demonstrated that mice with a deficiency of only one or two PAR bZip genes exhibit relatively mild phenotypes (5,7,8), whereas strong phenotypes are observed when all three PAR bZip genes are inactivated. Approximately half of these animals tend to have spontaneous and sound-induced epileptic seizures during the first three months following birth (5). Of note, locomotor activity appears to correlate with DBP levels (9). By contrast, DBP-deficient mice demonstrated reduced baseline locomotor activity and blunted behavioral responses to acute methamphetamine stimulation in an open field test.

The mitogen-activated protein kinase (MAPK) pathway is an extracellular signal pathway capable of causing a cell nuclear reaction and even activating components of the programmed cell death pathway, including extracellular signal-regulated protein kinase 1/2 (ERK1/2), ERK3/4, ERK5, p38 and c-Jun N-terminal kinase (JNK).

The MAPK pathway is widely expressed in the central nervous system (CNS). Each type of extracellular stimulus signal, including neurotransmitters, nerve trophic factors and growth factors, may use this pathway to affect synaptic transmission, neuronal remodeling, morphological differentiation and survival. Therefore, this signaling pathway is involved in the pathological processes associated with numerous types of nervous system disorders, including epilepsy. Based on the important physiological roles of DBP in the adult brain, it is important to examine whether DBP is abnormally expressed in IE and whether it may serve as a marker for the condition. MAPK and the signal pathways mentioned above have both been reported in epilepsy and correlated to the underlying mechanism of the disease. It may therefore be possible that DBP, through the interaction with MAPK, participates in the drug-resistant mechanisms of epilepsy. In order to examine the possible roles of DBP in the pathogenesis of IE, the present study detected DBP expression by immunohistochemically staining tissue from patients with IE and examined the correlation between DBP and MAPK by double-label immunofluorescence staining. It was hypothesized that DBP and MAPK have a key role in the mechanisms that underlie the pathogenesis of IE.

## Materials and methods

### IE group

In the present study, all of the patients with epilepsy had typical clinical manifestations and characteristic electroencephalograms. A total of 35 patients with IE were randomly selected from the epilepsy brain tissue collections of The First Affiliated Hospital of Chongqing Medical University (Chongqing, China). The diagnosis of seizure type was confirmed according to the 1981 International League Against Epilepsy. Prior to the surgery, the epileptic lesion was localized in all patients by brain magnetic resonance imaging (MRI) or computerized tomography (CT), and 24 h electroencephalography (EEG) or video EEG; sphenoidal electrode monitoring and intraoperative electrocorticography (ECOG) were performed to localize the epileptic lesion prior to resection in all patients. Two neuropathologists reviewed each case independently. The 35 patients were refractory to maximal doses of at least three AEDs, including phenytoin, valproic acid, carbamazepine, phenobarbital and topiramate. Brain MRI or CT, and the associated laboratory inspection, did not discover the presence of other nervous system diseases in any of the patients. Table I summarizes the patients’ clinical features. In these epilepsy patients, surgical removal of the epileptogenic zone in the temporal neocortex was an alternative treatment option. The pathological findings in the resected tissue included gliosis and neuronal loss, as well as signs of neuronal degeneration. Following lesion resection, the electrodes for intraoperative electrocorticography were placed on the remaining edge of tissue to ensure that the lesion was resected completely.

### Control group

For comparison, 15 histologically normal temporal neocortex samples were obtained from individuals treated for increased intracranial pressure due to head trauma. The time between mortality and surgery was <5 h. All of the patients were diagnosed, by the same two pathologists, with brain trauma and had no prior history of seizures or other neurological disorders. The majority of the neurological structural features appeared normal. Table II summarizes the clinical features of the controls.

The National Institutes of Health and the Ethics Committee on Human Research at Chongqing Medical University approved the present study (Chongqing, China). Informed consent was obtained from the patients or their relatives for the use of any data and tissues for the study.

### Tissue processing

The resected brain tissues were fixed in 10% buffered formalin. Following fixation in formalin for 24–48 h, the specimens were routinely paraffin-embedded, sectioned and preserved for future use at room temperature.

### Immunohistochemistry

Each paraffin-embedded sample was cut into 5-μm thick sections, which were spread on polylysine-coated slides. Each specimen produced 10 slices, of which one was selected randomly for immunohistochemical staining. Immunohistochemistry was performed according to the manufacturer’s instructions of the DAB Detection kit (Polymer, Zhongshan Golden Bridge, Inc., Beijing, China). First, the paraffin sections were deparaffinized, hydrated through graded alcohols and incubated in H_2_O_2_ (0.3% for 10 min). The sections were heated in a microwave oven for 20 min at 98°C in a citrate buffer (pH 6.0) for antigen retrieval, then cooled naturally to 37°C at room temperature and blocked in normal goat serum (1:10) for 10 min (Zhongshan Golden Bridge Inc., Beijing, China). The sections were incubated in primary rabbit anti-human DBP polyclonal antibody (1:200; Aviva Systems Biology Corporation, San Diego, CA, USA) overnight at 4°C, followed by a biotinylated anti-rabbit secondary antibody for 30 min at 37°C. Immunoreactivity was observed with 3,30-diaminobenzidine (DAB^+^; Zhongshan, Golden Bridge Inc.). Counterstaining was conducted with Harris hematoxylin. The blank controls were obtained by substituting the primary antibody with phosphate-buffered saline (PBS).

### Immunofluorescence

Each paraffin-embedded sample was cut into 10-μm thick sections, which were spread onto polylysine-coated slides. Each specimen produced 10 slices, of which one was selected randomly for analysis. The paraffin sections were deparaffinized, hydrated through graded alcohols and incubated in Triton (0.4% of 15 min). The sections were heated in a microwave oven for 20 min at 98°C in citrate buffer (pH 6.0) for antigen retrieval and then cooled naturally to 37°C at room temperature. Subsequently, the sections were blocked in calf serum mix followed by normal goat serum for 5 h (Zhongshan Golden Bridge Inc., Beijing). The sections were incubated in primary rabbit anti-human DBP polyclonal antibody (1:100; Aviva Systems Biology Corporation) and mouse anti-human MAPK polyclonal antibody (1:75; Zhongshan Golden Bridge Inc.) overnight at 4°C, followed by tetramethylrhodamine-conjugated AffiniPure goat anti-mouse immunoglobulin (Ig)G (1:50; Zhongshan Golden Bridge Inc.) and fluorescein isothiocyanate-conjugated AffiniPure goat anti-rabbit IgG (1:50; Zhongshan Golden Bridge Inc.) for 3 h (under protection from light) at room temperature. The sections were mounted with 50% glycerol and 50% PBS. The fluorescent-stained sections were observed by confocal microscopy (TCS SP2; Leica Microsystems GmbH, Wetzlar, Germany).

### Statistical analysis

Data are expressed as the mean ± standard deviation. Student’s t-test (SPSS version 17.0; SPSS, Inc., Chicago, IL, USA) was used for statistical analysis of the differences between the IE group and control group in humans.

## Results

### Demographic and clinical characteristics of the patients

The mean age of the patents with drug-refractory IE was 34.97±17.26 years and consisted of 12 male and 23 female patients. A total of 48.5% of patients had at least a 10-year history of seizure recurrence and 31.4% had a clinical history of epilepsy of >15 years. The control group had a mean age of 30±12.39 years and consisted of nine male and six female subjects. There were no significant differences in age or gender between the IE and control groups (P>0.05).

### Significant DBP staining in the temporal neocortex of patients with IE

**Figure 1:**
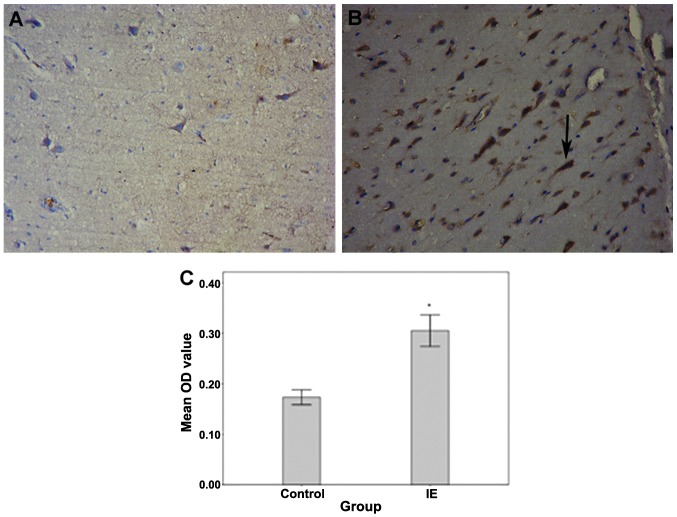
Immunohistochemistry with DBP in the temporal neocortex of the (A) epilepsy and (B) control subjects. The mean absorbance of DBP expression in the IE group was 0.31±0.03, whereas in the control group, it was 0.18±0.02 (P<0.05) (C). The difference between the mean OD values of the IE and the control groups was significant (^*^P<0.05). The arrow indicates a positively labeled neuron. DBP, D site of albumin promoter binding protein; IE, intractable epilepsy; OD, optical density.

**Figure 2:**
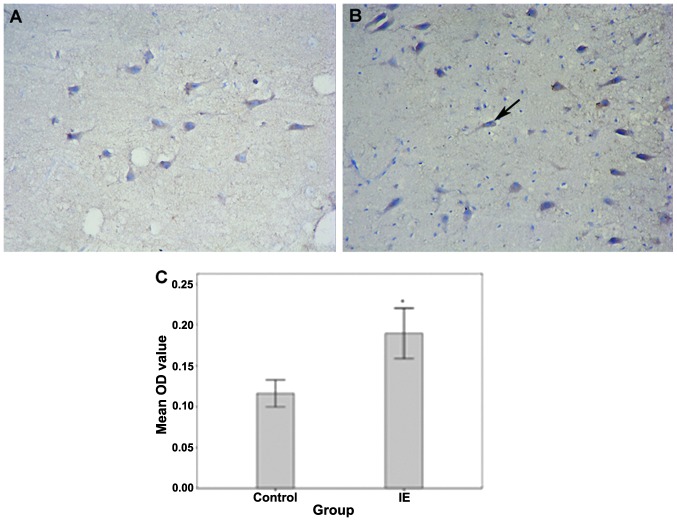
Immunohistochemistry with MAPK in the temporal neocortex of the (A) epilepsy and (B) control subjects (magnification, ×400). The mean absorbance of MAPK expression in the IE group was 0.19±0.03, whereas in the control group, it was 0.12±0.02 (P<0.05). (C) The difference between the mean OD values of the IE and the control groups was significant (^*^P<0.05). The arrow indicates a positively labeled neuron. MAPK, mitogen-activated protein kinase; IE, intractable epilepsy; OD, optical density.

**Figure 3:**
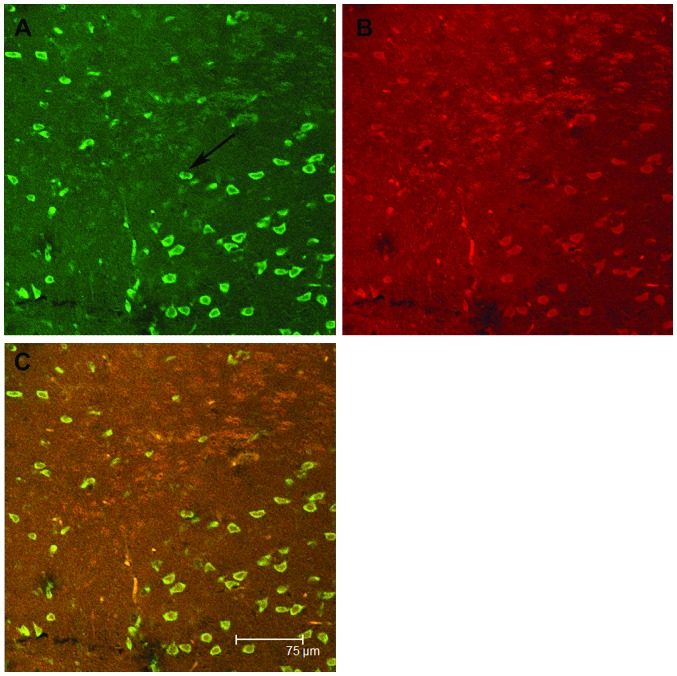
Immunofluorescence with DBP and MAPK in the temporal neocortex of the IE group (magnification, ×400). (A) DBP-stained neurons are green and (B) MAPK-stained neurons are red. (C) DBP and MAPK expression overlapped. The arrow indicates a positively labeled neuron. DBP, D site of albumin promoter binding protein; IE, intractable epilepsy; MAPK, mitogen-activated protein kinase.

In the IE group, DBP was consistently observed in all cases, whereas reduced DBP levels were apparent in the control group (Fig. 1). The expression of DBP was more apparent in the neurons of IE patients than in those of the control group and it was almost absent in glial cells in both groups. No immunoreactivity was observed in the negative control sections, in which the primary antibody was omitted. DBP expression was significantly higher in the brain tissues of patients with IE as compared with that in the control group. The mean absorbance of DBP expression in the IE group was 0.31±0.03, whereas in the control group, it was 0.18±0.02 (P<0.05). The expression of MAPK was upregulated in IE patients as compared with that in the controls (Fig. 2). The mean absorbance of MAPK expression in the IE group was 0.19±0.03, whereas in the control group, it was 0.12±0.02 (P<0.05). Immunofluorescence staining showed that in the IE group, the expression of both DBP and MAPK was present in the same neurons, whereas almost no immunoreactivity was apparent in the glia from either group (Fig. 3).

## Discussion

Epileptic seizures are characterized by high-frequency perturbations of the activity of cells of the CNS, which may subsequently initiate a series of complex nervous biological effects. Each type of intranuclear adverse factor causes a reaction through a series of signal transduction pathways. From that moment, activated signaling molecules may develop continuous cascade reactions, changing the expression of numerous genes, exhibiting durability functions and finally, causing delayed nerve cell death. The pathological mechanisms involved in IE are intricate and, to date, not fully understood. Numerous studies have reported that DBP and MAPK are associated with the pathogenesis of epilepsy. However, it is not clear whether DBP, and its interaction with MAPK, participate in mechanisms of drug resistance in epilepsy. The aim of the present study was to clarify the role of DBP and MAPK in the mechanisms that underlie the pathogenesis of IE.

Immunohistochemical analysis of protein levels demonstrated that DBP expression was significantly higher in the brain tissues of patients with IE and that it was localized to neurons specifically, while nearly absent in glia. It is therefore concluded that DBP does not participate in gliosis but exerts its biological effect through neurons, contributing to the mechanisms that trigger epilepsy.

Several studies have suggested that PAR bZip triple-knockout mice have high sensitivity to lethal sound-induced and spontaneous seizures that are immediately followed by a disturbance of neurotransmitter homeostasis (5). DBP-deficient mice do not exhibit such major abnormalities and this is possibly due to functional redundancy (10). The phenotype is considered to be caused by a decrease in pyridoxal kinase (Pdxk), which is encoded by a PAR bZIP target gene in the liver and the brain. Pyridoxal kinase converts vitamin B6 derivatives into pyridoxal phosphate (PLP), the coenzyme of numerous enzymes involved in amino acid and neurotransmitter metabolism. PLP also serves as the coenzyme of aromatic amino acid decarboxylase, an enzyme involved in the synthesis of the monoamines serotonin and dopamine (11).

These neurotransmitters have been associated with the occurrence of epileptic events. Even moderate reductions in the concentration of PLP have been demonstrated to provoke epileptic seizures (12,13). During *et al* (13) suggested that DBP downregulation paralleled anticonvulsive effects following glucagon-like peptide-1 receptor GLP-1R activation, which follows abnormally reduced susceptibility to kainate-induced seizures. The present study suggested that DBP levels in the brain may impact the modulation of seizures. Matthias’ microarray analysis demonstrated that hippocampal activation of GLP-1R, which is associated with improved learning and neuroprotection, resulted in the suppression of DBP (6). By contrast, the other two PAR bZip transcription factor family members, TEF and HLF, did not produce evident changes. The present study also demonstrated a compensatory shut-down of DBP expression to counteract neuronal overexcitation through glutamate receptors. However, it is unclear whether persistent upregulation of DBP increases the sensitivity to epilepsy. Studies have demonstrated that the persistent upregulation of DBP may cause the upregulation of pdxk and induce the activation of ERK1/2 and MAPK, as compared with the controls (6). The ERK1/2 signaling pathway belongs to the MAPK family and its major roles involve coordinating neuronal responses to external signaling, affecting the remodeling of synapses, axonal growth, long term potentiation and neuronal excitability, by regulatory gene expression and protein synthesis (14). The continual activation of this pathway prompts persistent delivery of neurotransmitters. The latter possibly maintains the activation of the ERK circuit through phosphorylation, and ERK activation subsequently enhances the delivery of synaptic transmitters through positive feedback.

MAPK expression has been primarily observed in neurons following seizures and the present results confirmed that while the upregulation of MAPK was expressed in glial cells and neurons of both groups, it was mainly observed in neurons from patients with IE. This suggests a significant role of MAPK in neurons associated with chronic epilepsy. In the liver, DBP has an important role in detoxification and drug metabolism. Epilepsy causes the upregulation of DBP and MAPK, and while MAPK participates in the progression and promotes the development of epilepsy, DBP activates MAPK through positive feedback (15,16).

In conclusion, the present study demonstrated that the expression of DBP and MAPK in patients with IE was upregulated. The simultaneous co-expression of DBP and MAPK indicated that the interaction of DBP and MAPK may participate in the remodeling of synapses and the drug resistance mechanism of epilepsy. However, it remains elusive how DBP and MAPK participate in this mechanism. Further studies are required to determine the role of DBP and MAPK in the mechanism underlying the pathogenesis of IE, and the present study offers a new direction of inquiry into this concept.

## Figures and Tables

**Table I:** Clinical characteristics of temporal lobe epilepsy patients.

Patient no.	Gender	Age (years)	Duration (years)	Seizure type	AEDs	Resection tissue	Pathology
1	M	28	2	SGS	CBZ, PHT, VPA	TNr	nd
2	F	36	16	SGS	CBZ, PHT, VPA, PB	TNl	nd, nl
3	F	22	3	SGS	CBZ, VPA, PHT	TNr	nd, nl, g
4	M	15	9	SGS	CBZ, PHT, VPA	TNl	g
5	M	14	7	CPS	CBZ, PHT, PB	TNl	nd, g
6	M	20	10	SGS	CBZ, PHT, VPA′	TNr	nd, nl, g
7	F	17	5	CPS	CBZ, PB, CLB	TNr	nd, g
8	F	58	40	CPS	CBZ, PHT, CLB	TNl	nd, g
9	M	8	5	CPS	CBZ, PB, VPA	TNl	nd, g
10	M	18	13	SGS	CBZ, PRM, PHT	TNr	nd, g
11	M	15	4	SGS	CBZ, PHT, TPM	TNr	nd, g
12	M	32	20	SGS	CBZ, VPA, TPM	TNl	nl, g
13	F	47	18	CPS	CBZ, PB, CLB, PRM	TNr	nd, nl, g
14	M	41	20	SGS	CBZ, PHT, VPA, PB	TNr	nd, nl, g
15	M	17	5	SGS	TPM, VPA, PHT	TNr	nd, nl, g
16	M	22	16	SGS	CBZ, TPM, VPA, PB	TNl	nl, g
17	M	16	14	SGS	CBZ, PHT, PB	TNl	nl, nd, g
18	M	15	15	SGS	CBZ, CLB, TPM	TNl	nd, g
19	M	25	1	SGS	CBZ, PRM, TPM, PB	TNr	nd
20	M	26	9	SGS	CBZ, PHT, VPA, CLB	TNr	nl, nd, g
21	F	45	3	CPS	CBZ, VPA, TPM	TNr	g
22	M	26	14	SGS	CBZ, TPM, VPA, CLB	TNr	nl, nd, g
23	M	22	21	SGS	CBZ, VPA, TPM, PB	TNl	nd, g
24	M	22	12	SGS	CBZ, VPA, PRM, NIM, PB	TNr	nl, nd, g
25	M	12	3	SGS	CBZ, VPA, PB, CLB, PHT	TNr	nl, nd, g
26	F	15	2	CPS	CBZ, TPM, VPA	TNl	nd, g
27	M	22	1	CPS	CBZ, PB, TPM	TNr	nl
28	M	15	2	SGS	CBZ, VPA, PHT	TNr	nd, g
29	M	24	22	SGS	CBZ, VPA, PB, TPM, CLB	TNl	nl, nd, g
30	F	22	5	SGS	CBZ, VPA, CLB	TNr	nl
31	F	18	16	SGS	CBZ, PB, VPA	TNl	nd, g
32	F	23	8	SGS	CBZ, PB, TPM	TNl	nl
33	M	16	8	SGS	CBZ, VPA, TPM	TNl	g
34	F	44	12	CPS	CBZ, TMP, VPA	TNl	nd, g
35	F	24	21	SGS	CBZ, PB, PRM, VPA	TNr	nl, g

AEDs, antiepileptic drugs; CBZ, carbamazepine; PHT, phenytoin; VPA, valproic acid; PB, phenobarbital; TPM, topiramate; CLB, clonazepam; PRM, primaclone; NIM, nimodipine; TN, temporal neocortex; nl, neuronal loss; nd, neuronal degeneration; g, gliosis; SGS, secondarily generalized seizure; CPS, complex partial seizure; l, left; R, right; F, female; M, male.

**Table II:** Clinical characteristics of control patients.

Gender	Age (years)	Etiological diagnosis	Resection tissue	Adjacent tissue pathology
Male	47	Trauma	TNl	Normal
Female	22	Trauma	TNl	Normal
Male	31	Trauma	TNl	Normal
Male	23	Trauma	TNr	Normal
Female	20	Trauma	TNr	Normal
Male	46	Trauma	TNl	Normal
Male	21	Trauma	TNr	Normal
Male	29	Trauma	TNl	Normal
Female	42	Trauma	TNr	Normal
Male	38	Trauma	TNl	Normal
Male	38	Trauma	TNl	Normal
Female	17	Trauma	TNr	Normal
Male	48	Trauma	TNl	Normal
Female	18	Trauma	TNr	Normal
Female	10	Trauma	TNl	Normal

TN, temporal neocortex; l, left; r, right.
